# Book Review

**Published:** 1926

**Authors:** 


### Reviews of Books.

**The Diagnosis and Treatment of Tuberculosis of the Hip.** By G. R. Girdlestone, B.M. (Oxon.), F.R.C.S. Pp. x., 94. Oxford Medical Publications. 1925. Price 8s. 6d. — The author writes with freshness, originality and conviction which immediately claim attention. The book is entirely clinical in its scope and practical in its design. The importance of early diagnosis is insisted upon, and the view is expressed that all doubtful cases, even with negative X-ray findings, should be sent to a special institution for further investigation and treatment. This may be an unattainable council of perfection, but it is well to have it set up as a standard. The X-ray figures and the typical case reports are most instructive. The chief criticism which may be made is that the book is too small to deal even with the practical aspect of the question, and yet appears so much to be a specialist's monograph that it will not be read by the general practitioner.

**Manipulative Surgery: Principles and Practice.** By A. G. Timbrell Fisher, F.R.C.S. (Eng.). Pp. viii., 168, with 62 illustrations. London: H. K. Lewis & Co. Ltd. 1925. Price 7s. 6d.—The author considers it desirable to draw the attention of the medical profession to the possibilities of manipulative surgery. It is popularly supposed that unqualified "bone setters" have possession of knowledge and methods which are denied to the medical profession. Probably the better-class "bone-setter" would rather claim special dexterity and instinct than special knowledge. A short historical introduction serves to give the views of such leading men as Hunter, Hilton, Thomas, Wharton Hood and Paget, and a strong opinion is expressed in favour of special teaching of manipulative surgery in the medical curriculum. The cases said to be cured by bone-setters are: (1) adhesions in and round joints; (2) functional conditions; (3) unreduced dislocation or sub-luxations. In the matter of diagnosis of conditions likely to be benefited by manipulation stress is laid upon evidence of active disease, and especially tubercle. The body of the book consists of a description of the anatomy, movements and disabilities of the main joints, a consideration of the knee occupying the largest space. The illustrations are clear and good, though it may be questioned whether many of those depicting normal limbs are necessary. Mr. Fisher, who has already done good work on internal derangement of the knee joint, is to be congratulated on having given an accurate and concise account of a subject which is too often shrouded in mystery.

**The Early Diagnosis of the Acute Abdomen.** By Zachary Cope, B.A., M.D., M.S. (Lond.), F.R.C.S. Pp. x., 233. Third Edition. Oxford Medical Publications. 1925. Price 10s. 6d.—The appearance of a third edition of this book is the best proof that it has filled a want. While it is no doubt true that the best place to learn the subtleties of abdominal and every other kind of diagnosis is the bedside, there are many occasions when the inquirer, especially if he be inexperienced, will turn with gratitude to such a mine of information as is contained in the book under notice. There is no finality about abdominal diagnosis, and mistakes are made by those even of the ripest experience. Most of these errors are the result of the failure to observe some point which is perfectly obvious to the seeing eye. In so far as books can teach so essentially clinical a subject as abdominal diagnosis, we can imagine no book more helpful than this. Think of any case where your diagnosis was wrong, and here you will surely find what would have put you on the right track if only you had had it in your mind. One or two examples must suffice. The confusion of cases of right-sided chest disease with appendicitis is well known, but mistakes are still made only too often. A more subtle error, the confusion of "abdominal" uræmia with surgical conditions, still constitutes a trap for the unwary. Both these points, and countless similar ones, receive adequate notice. We have no doubt but that the new edition will receive a welcome no less warm than its predecessors. The student, qualified or unqualified, who absorbs the contents of this book and applies his knowledge at the bedside will avoid many a pitfall which might otherwise have proved his undoing.

**Coaching Manual for Midwifery Students.** By Félicic Norton, S.R.N. Pp. viii., 110. London: The Scientific Press. 1925. Price 1s. 6d. net.—A small revision manual for midwives, especially useful for reading between the written and oral examinations of the Central Midwives Board.

**A Synopsis of Gynæcology.** By Arthur Gray, F.R.C.S., M.R.C.P. Pp. viii., 352. London: E. Arnold & Co. 1925.

Price 10s. 6d. net.—This, another of the " Synopsis " Series, is an extremely well-arranged text-book. It contains not only all the essentials of the various gynæcological conditions, but also excellent chapters on the endocrine organs in their relation to diseases of women, and on forensic gynæcology. The inclusion of this last chapter is extremely useful, and should be invaluable to the newly-qualified doctor. This text-book should be in the possession of every student, to be read especially for revision prior to his examination.

**Public Health Laboratory Work (Chemistry).** By HENRY R. KENWOOD, C.M.G., M.B., F.R.S. (Edin.). Pp. xi., 369. Eighth Edition. London: H. K. Lewis & Co. Ltd. 1925. Price 12s. 6d. net.—We welcome a further edition of this well-established text-book of Public Health Laboratory Work, which is mainly confined to the chemical side, but is also interspersed with a certain amount of bacteriology. This edition adheres to the main lines of its predecessors, and, with those few additions which bring the book up to date, constitutes the best text-book on Public Health Chemistry for the examination for the Diploma in Public Health, while it is also an exceedingly valuable book for reference purposes. In Part 1 a chapter dealing with the opinion on water samples gives an excellent series of varying types of potable water, good, bad and indifferent, which will be of great value to the student. Part 2 appears as merely an addendum to this part, and might almost have been included in it as a separate chapter: it would then have been unnecessary to repeat a description of Winkler's process. Part 3 deals with air and the forms of pollution to which air is liable. Part 4 (food examination) is that section of the book which has most tendency to grow, and those engaged in the teaching of Public Health Chemistry know how difficult it is to confine this portion of the subject within reasonable limits. A remark on page 207 is so apt that we will quote it: " Whether milk is naturally poor or has been made so by the addition of water, the dairyman who sells it defrauds the purchaser, for the latter demands and pays for pure milk of average quality." This point was well illustrated in Bristol recently, when a cow was discovered in a herd which yielded milk equivalent to milk of average quality after 7 per cent. of water had been added. We think the volatile acids in butter should have been dealt with by the Reichert-Polenske process, with merely a reference to the Reichert-Meissel method, as the latter is now rarely carried out. We are glad to see the inclusion of the new standard for condensed milk, though the analysis of this substance involves work which is far beyond the scope of the average Public Health course. This part contains a description of the analysis of Rag Flock, and the book concludes with a short part on disinfectants. It is probably correct to say that the majority of doctors at present engaged in the Public Health service were brought up on Kenwood's *Public Health Laboratory Work*, and we look forward to the day when a resuscitated Public Health Course in the University may bring this excellent book into use.

**The Extra Pharmacopœia of Martindale and Westcott.** Revised by W. H. Martindale, Ph.D., Ph.Ch., F.C.S., and W. Wynn Westcott, M.B. (Lond.), D.P.H. Eighteenth Edition. Volume II. Pp. xiii., 728. London: H. K. Lewis & Co. Ltd. 1925. Price 20s. net.—This volume deals chiefly, as in previous issues, with analytical, experimental and research work, Volume I. being concerned largely with matters relating to treatment. It would be quite impossible in a short review to deal with all the subjects referred to and the analytical methods described, but the fact that the book is well up to date is illustrated quite well by the inclusion of an account of the work on Isotopes and Atomic Numbers carried out by Aston and others. Those practitioners of medicine who are interested in pharmacological problems will read with interest details relating to the determination of activity of certain well-known drugs, notably aspirin and its derivatives and digitalis preparations. There is an interesting section on the organic arsenic compounds, in which manufacturing and analytical methods are dealt with and which includes a short historical account of Salvarsan by Ehrlich. The analytical memoranda contain descriptions of methods used in blood and urine analysis and in the investigation of stomach contents. Some instructive references are made to proprietary medicines, and the alleged composition of many of these is given. Under "Bacteriological Notes" are included a vast number of different subjects, among these being summaries of work on researches relating to the causation of malignant tumours; food poisoning is also dealt with in this section, including Botulism and canned food poisoning. A considerable amount of space is naturally given under this heading to the staining and differential recognition of bacteria, and a number of useful formulæ are given. The penultimate section includes glossaries of Arabic, Belgian, Danish, Dutch, French, German, Italian, Portuguese and Spanish terms, and the volume ends with an alphabetical index and posological table. A most valuable feature of this volume is that many references are given to original papers, which is a great advantage to workers in special branches of research. We congratulate authors and publishers alike on the excellence of their work, and consider that it should be in the library of all those medical men who are interested in the scientific progress of their profession.

**Post-Mortem Appearances.** By Joan M. Ross, M.B., B.S. (Lond.), M.R.C.S., L.R.C.P. Pp. 216. Oxford University Press. 1925. Price 7s. 6d. net.—This little book of 216 pages in convenient pocket size contains in a condensed but very lucid form a description of the post-mortem appearances in the commoner diseases. Following some general remarks, which include directions for carrying out a post-mortem examination and methods of preserving specimens, the author describes the post-mortem findings in death from physical and chemical agents, and from general and systemic diseases. Information as regards normal weights and measurements, date of ossification centres, etc., are given in an appendix. The book will be very useful to the student, since it contains much that is not mentioned in the ordinary text-books, whilst the practitioner will find it a ready reference book for the successful carrying out of an occasional post-mortem examination.

**The Post-Graduate Medical Journal.** The Official Organ of the Fellowship of Medicine. 1 Bedford Street, London, W.C. 2. Volume I. No. 1. October, 1925. Price 6d. Annual Subscription 6s.—*The Post-Graduate Journal* is the official organ of the Fellowship of Medicine. It concerns itself chiefly with the arrangements for post-graduate work in London, but in time, no doubt, it will be extended so as to include all the provincial post-graduate facilities. In addition, the Editor proposes to publish a selection of the lectures which are delivered at the instance of the Fellowship of Medicine. "It will be our endeavour," he says, "to select only such subjects as will from their essentially practical character make the strongest appeal to the general practitioner for whom the resources of post-graduate teaching are particularly indicated." The first number contains an excellent contribution by Sir Humphry Rolleston on the "Medical Aspects of Gallstones," and a concise consideration of "The Prevention of Puerperal Sepsis," by Dr. T. W. Eden. Both of these are of a practical nature, and none of our readers should miss consulting them. We extend a hearty welcome to the new venture, and wish it success. The medical literature of to-day is an increasing burden for the graduate, but if post-graduate work is to be systematised, made attractive and available, a periodical such as this should be of considerable use in directing the intending worker where and how he may best spend his scanty time for keeping abreast with current developments.

**An Index of Treatment.** By VARIOUS WRITERS. Edited by ROBERT HUTCHISON, M.D., F.R.C.P., and JAMES SHERREN, C.B., F.R.C.S. Ninth Edition, revised and enlarged. Pp. xviii., 1,035. Bristol: John Wright & Sons Ltd. 1925. Price 42s.—Four years have elapsed since the last edition of this valuable reference work appeared; during that time many important advances have been made in the science and practice of medicine in all its branches. The ninth edition now under review embodies all of these advances. For example, the discovery of Insulin has revolutionised the treatment of diabetes entirely. The directions for its use will be found clearly set out. Our knowledge of asthma, especially of the forms due to protein sensitisation, has been considerably enlarged: this forms the subject of a re-written and instructive article. The progress made in the treatment of fractures is described very fully. There are many additions to and alterations in the articles dealing with gonorrhœa, nephritis, syphilis and tropical diseases. The surgery of the prostate, kidney and gall-bladder has developed also in the past few years, and these developments are duly noted. It is unnecessary to recommend such a well-known work to our readers; it is sufficient to say that it has been brought up to date in the ninth edition.

**The Stomach and Upper Alimentary Canal in Health and Disease.** By T. IZOD BENNETT, M.D. (Lond.), M.R.C.P. Pp. xv., 344. London: William Heinemann (Medical Books) Ltd. 1925. Price 21s. net.—Dr. Izod Bennett has written a book of great importance as well as interest to the general practitioner. He is especially deserving of thanks for the inclusion of a chapter on diseases of the teeth and gums, which helps to a clearer understanding of the overworked phrase "oral sepsis." As the author says, "it is only by the medical practitioner's familiarising himself with the rudiments of dental pathology, and keeping in close touch with his patients' dental surgeons, that the necessary co-operation will be secured which will eventually lead to a great improvement in the health of the community." Dr. Bennett displays well-balanced judgment in his remarks on the part played by oral infection in the production of general diseases. The physiology of the stomach is most readably described, and the blending of clinical application with physiological observation is admirable. The examination of patients is given in very detailed fashion, without being at all long-winded. The explanations of gastric analyses and of radiographic appearances are most understandable ; and Dr. Bennett is extraordinarily level-headed in appraising the value of the various aids to diagnosis. His account of peptic ulcers, their symptoms, diagnosis, treatment and complications is as good as any we have read. He is a staunch advocate of Sippy's method of dieting, "its main principles," he says, "should be rigidly adhered to," although "it was designed for the American patient, and items must be changed to adapt it for the British palate." The advice given as to the choice of cases for operative treatment is thoroughly sound. Another chapter worthy of careful attention is that on "Disorders of gastric function." This book is a notable contribution to medical literature, it brings recent advances in physiology and pathology into close touch with the everyday problems which confront the practitioner. The diagrams and illustrations are well chosen and clearly reproduced. We have praised the book highly, but not more than it deserves.

**Selected Papers, Surgical and Pathological.** By F. T. Paul, Ch.M. (Liverpool), F.R.C.S. Eng. Pp. vii., 284. London: Baillière, Tindall & Cox. 1925. Price 15s.—This volume, containing reprints of Mr. Paul's more important contributions to surgical and pathological literature, is the outcome of the desire of the surgeons of Liverpool to express their admiration and appreciation of the quality of Mr. Paul's work, and as a token of their respect and affection on the occasion of his seventy-fifth birthday. "Paul's Tubes" for colotomy and colectomy are familiar to all surgeons, and frequently used. They are a constant reminder of the fact that when the technique of colectomy was in a very imperfect stage of evolution Mr. Paul introduced a method of operating which was much safer and more successful than any other then in use. And it is interesting to read in this book the original paper, published in 1892, which contains the earliest description by the author of his method. We have not space to comment on the various addresses and papers gathered together here, but must content ourselves by saying that each well repays attentive study. They are full of penetrating observation and practical wisdom.

**A Textbook of Pathology, General and Special.** By J. MARTIN BEATTIE, M.A. (N.Z.), M.D. (Edin.), M.R.C.S., L.R.C.P. (Lond.), and W. E. CARNEGIE DICKSON, M.D., B.Sc., F.R.C.P. (Edin.). With 499 illustrations in the text and 17 coloured plates from original preparations. Third Edition. Royal 8vo. Pp. 1,130. London: Wm. Heinemann (Medical Books) Ltd. Complete Volume, price 42s. net. Or in Two Volumes: Part I., General Pathology; Part II., Special Pathology, 25s. net each.—In our recent review of the third edition of this well-known text-book (vol. xlii., p. 239) we commented unfavourably on the great weight of its one volume form. Our attention has since been drawn to the fact that the work may be obtained either in one volume or in a two volume form as indicated above. Obviously our objection to the weight of the one form does not apply to the other.

**History of Medicine.** By Dr. MAX NEUBURGER. Translated by ERNEST PLAYFAIR, M.B., M.R.C.P. Volume II. Part I. Pp. vii., 135. Oxford Medical Publications. 1925. Price 7s. 6d.—The publishers' note to this first part of the second volume expresses regret that it is not yet possible to complete the English edition of Neuburger's *History of Medicine.* The present instalment is issued without waiting for the remaining parts, which will follow as and when the German original is published. A work such as Neuburger's is one which demands time as well as unremitting industry. The patience which Neuburger has shown in first-hand study of his sources will explain to every reader of his *History* why the publication is slow. Neuburger has accepted little or nothing on the authority of others. He views each period through his own eyes and draws his conclusions from the extant writings of the period in question. This section deals with medicine from the early Middle Ages, about the sixth century up to the later Middle Ages, closing just before the revival of learning in the fifteenth century. Neuburger traces with vivid realism the causes which led to the gradual transference of scientific study to ecclesiastical circles, showing how Italy, once the centre of civilisation and learning, was overspread by "havoc and devastation, famine and misery, savagery and brutality" during the sixth century. Finally, science "deprived of almost every other aid . . . was forced to betake itself to the shelter of the Church and to seek refuge in monastic seclusion." The price of the Church's protection to science in that age was the linking of superstition, thaumaturgy and theurgy with the traditions of rational practice in medicine. In Italy there survived, however, some remnant of antique heathen culture independent of and even in opposition to the Church, awaiting better and quieter days. Neuburger points out that beyond the Alps clergy and monks, in medicine as in culture in general, occupied a far more dominant position than in Italy. The part played by the Celtic monasteries is admirably described : "It appears a wonderful dispensation of Providence that, during the terrible devastations of the sixth to eighth centuries, in a truly forlorn age of general decay of civilisation the heirlooms from antiquity should have been hidden in Ireland, the *Ultima Thule* of Christianity." Alcuin's influence on Charlemagne meets with full recognition, for it was mainly through his influence that the scientific traditions of the schools of York, Winchester and Canterbury were disseminated upon the Continent. Thus, from the ninth century onwards, medicine was included in the educational system of the Frankish monastic schools. In the eleventh and twelfth centuries the famous school of Salerno was in its prime, a health resort for invalids and a rallying-point for physicians who perhaps had not altogether lost the threads of late-Roman tradition. Here a school grew somehow and flourished without an ecclesiastical foundation, owing something, no doubt, to the Graeco-Roman tradition, but more perhaps to the Arabians with their Jewish and Nestorian physicians. It was at Salerno that the art of surgery was re-established on a scientific basis. In the Christian West the rule held good : "Ecclesia abhorret a sanguine," and surgery was relinquished to uneducated empirics. To Salerno may be traced the rise of schools of medicine at Montpellier, Bologna and Paris. Thus at last the spark of learning which the Arabians had preserved was rekindled in the West. The remainder of this volume traces clearly and fully the gradual rise of importance of the Arabian tradition until the dawn of the Renaissance is reached. Neuburger's grasp of the spirit of the age is unrivalled, he tells his story with rare artistry, and, in this English version, he owes not a little to his admirable translator.

